# Effectiveness and cytokine profile of combined anti-vascular endothelial growth factor and corticosteroid therapy for chronic retinal vein occlusion

**DOI:** 10.1007/s00417-025-06738-4

**Published:** 2025-01-18

**Authors:** Yusuke Arai, Hidenori Takahashi, Satoru Inoda, Shinichi Sakamoto, Hidetoshi Kawashima, Yasuo Yanagi

**Affiliations:** 1 Department of Ophthalmology, Jichi Medical University, 3311-1 Yakushiji, Shimotsuke, 329-0498 Tochigi Japan; 2 Department of Ophthalmology and Micro-Technology, Yokohama City University, 4-57 Urafunecho, Minami-ku, Yokohama, 232-0024 Kanagawa Japan

**Keywords:** Chronic retinal vein occlusion, Combination therapy, Corticosteroids, Anti-VEGF, Intraocular inflammatory cytokines

## Abstract

**Purpose:**

To investigate whether sub-Tenon injection of triamcinolone acetonide (STTA) combined with anti-vascular endothelial growth factor (VEGF) prolongs the recurrence intervals of macular edema (ME) for chronic retinal vein occlusion (RVO) and to investigate the differences in intraocular inflammatory cytokines between good responders (GRs) and non-responders (NRs).

**Methods:**

This retrospective, observational study involved 42 eyes of 42 patients with ME due to chronic RVO who had received only anti-VEGF for ≥ 1 year and were transitioned to combination therapy. GRs were defined as patients whose recurrence intervals were prolonged by ≥ 2 weeks compared with patients receiving anti-VEGF alone. Moreover, immediately before starting the combined therapy, aqueous humor was collected and the following inflammatory cytokines were compared between GRs and NRs: CCL11, MCP-3, IP-10, CCL13, G-CSF, GM-CSF, IL-1α, IL-15, IL-4, M-CSF, MMP-9, TNF-α, MCP-1, CXCL-1, CXCL12, IL-8, galectin-1, IFN-γ, IL-12, IL-2, IL-6, MMP-1, PDGF-AA, and VEGF-A. These results were analyzed by nominal logistic regression after stepwise variable selection.

**Results:**

There were 26 eyes (62%) in the GR group. Nominal logistic analyses showed that a higher concentration of IL-1α (*P* = 0.016) and lower concentrations of IL-5 (*P* = 0.015), IL-6 (*P* = 0.022), and galectin-1 (*P* = 0.015) were significantly associated with the extension of the time from injection to recurrence of ME.

**Conclusion:**

Combined anti-VEGF and STTA therapy for chronic RVO was effective in 62% of patients, suggesting the effectiveness of STTA. Higher IL-1α and lower IL-5, IL-6, and galectin-1 were the factors associated with combined treatment effectiveness.

## Key Messages

*****What is known***:**

- Treatment of chronic retinal vein occlusion (RVO) is burdensome for patients, with about 50% of patients still requiring anti-vascular endothelial growth factor therapy 4 years after the first treatment.

*****What is new***:**

- We investigated whether sub-Tenon injection of triamcinolone acetonide (STTA) combined with anti-vascular endothelial growth factor (VEGF) prolongs the recurrence intervals of macular edema (ME) for chronic retinal vein occlusion (RVO). 
- Combined anti-VEGF and STTA therapy for chronic RVO was effective in 62% of patients. Higher IL-1α as well as lower IL-5, IL-6, and galectin-1 were the factors associated with combined treatment effectiveness.

## Introduction

Retinal vein occlusion (RVO) is a retinal vascular disease that causes significant vision loss due to refractory macular edema (ME) [1, 2]. Increased inflammatory mediators and upregulated vascular endothelial growth factor (VEGF) contribute to the vascular leakage, breakdown of the blood–retinal barrier, and ME seen in RVO [3, 4]. Large clinical trials have demonstrated the efficacy of anti-VEGF therapy in RVO [5–7], and anti-VEGF therapy is currently used as the first choice of treatment and is the standard of care for ME secondary to RVO [8, 9].

The RETAIN study reported long-term benefits of anti-VEGF drugs for RVO [10]; however, frequent intravitreal ranibizumab injections were required in about half (56%) of the patients within 4 years after the first treatment. Another clinical study reported that frequent injections (an average of 5.5 injections per year) were still required even after 5 years [11]. Thus, continuous anti-VEGF therapy imposes considerable financial and time burdens on patients. We previously demonstrated that personalized proactive dosing methods [12, 13] may reduce the burden; however, long-term treatment involving frequent injections was still necessary for about half of the patients. Accordingly, RVO chronicity is a major problem, and it has been suggested that a vicious cycle of inflammation may be involved in the background of chronic RVO [3, 4].

The efficacy of corticosteroids in treating ME due to RVO has been demonstrated by two major trials: SCORE and GENEVA [14–16]. However, corticosteroids are not used as a first line treatment because the risk of complications such as elevated intraocular pressure (IOP) is too high. Previous studies suggested that sub-Tenon triamcinolone injection (STTA) given as an adjunct therapy in combination with anti-VEGFs might be effective in reducing the number of anti-VEGF injections for ME due to branch retinal vein occlusion (BRVO) [17]and reducing ME in central retinal vein occlusion (CRVO) [18]. Complications such as cataract and IOP spike appear to be less frequent with STTA than with intravitreal corticosteroid injection. We hypothesized that, because both inflammation and VEGF might have strong effects on chronic RVO, anti-VEGF injection alone is not effective enough to resolve it. Previous reports have shown that inflammatory cytokines such as MCP-1, IL-6, and IP-10 are associated with ME in BRVO and CRVO [3, 4]. Also, given that corticosteroid therapy is classically used for inflammation, we decided to investigate whether combination therapy using corticosteroids and anti-VEGF injections might be more effective for chronic RVO. In addition, we examined differences in intraocular inflammatory cytokine profiles between good responders (GRs) and non-responders (NRs) and searched for inflammatory cytokine factors associated with GR.

## Methods

### Study design and approval

This retrospective and observational study investigated 42 eyes of 42 patients with refractory ME due to chronic RVO, including BRVO and CRVO, who had been treated solely with anti-VEGF injections for ≥ 1 year. The present study does not distinguish between ischemic and non-ischemic cases.

The study was approved by the Institutional Review Board of Jichi Medical University and adhered to the tenets of the Declaration of Helsinki. All patients provided informed consent prior to the procedures.

### Methods and ophthalmic examination

All patients had been treated solely with anti-VEGF injections for ≥ 1 year. No restrictions were placed on the type of anti-VEGF drug used. This study included three type of anti-VEGF drugs: aflibercept, ranibizumab, bevacizumab. We investigated whether the recurrence intervals of ME could be prolonged by anti-VEGF injections combined with STTA. A 27-G needle was passed through the inferior-lateral bulbar conjunctiva, and 20 mg (0.5 ml) triamcinolone acetonide (MaQaid^®^ Ophthalmic injection 40 mg; Wakamoto Pharmaceutical, Tokyo, Japan) was administered. The VEGF drug was the same type as that administered during VEGF drug monotherapy. GRs were defined as patients whose recurrence intervals were prolonged by ≥ 2 weeks compared with the period that the patients were on anti-VEGF injections alone. The recurrence period during the anti-VEGF only period was defined as the maximum duration of VEGF administered with the treat-and-extend method at 1-week intervals. Recurrence was defined as the presence of ME and/or serous retinal detachment on any macular OCT scan. OCT was performed using DRI OCT Triton (Topcon Corporation, Tokyo, Japan). The images were captured and evaluated by B-scan of 6 mm radial sections. In this study, OCTA and FA were not used for initial diagnosis.

The criteria for re-injection was the occurrence of even a mild relapse. In addition, at least two of the consecutive recurrence periods were the same. The combined therapy was performed immediately after its maximum duration. The next visit after combined therapy was set at the same number of weeks as the maximum period. If there was a recurrence at that time, it was defined as NR. If there was no recurrence, we would check again at 2-week intervals to see how long recurrence would take. After 3 months, we decided to see the patient every other month. When there was no recurrence, the patient was followed up for 1 year after combination therapy. ‘No recurrence’ was set as no recurrence within 1 year. Cases of cataract surgery within 3 years of combination therapy were defined as complications of STTA administration.

### Inflammatory cytokines

Immediately before starting the combined therapy, aqueous humor samples (approximately 0.2 mL) were collected by manual aspiration into disposable syringes through a clear corneal paracentesis under an operating microscope, before being immediately transferred to sterile tubes, snap-frozen, and stored at − 80 °C until needed. Cytokine concentrations in the aqueous humor samples were subsequently analyzed using a LUNARIS™ Human 11-Plex Ophthalmology Kit (AYOXXA Biosystem, Cologne, Germany). The following cytokines were measured: C-X-C motif chemokine ligand 1 (CXCL1), CXCL12, interferon-γ-inducible protein 10 (IP-10), C-C motif chemokine ligand 2 (CCL11), CCL13, interleukin 1α (IL-1α), IL-2, IL-4, IL-6, IL-8, IL-12, IL-15, matrix metallopeptidase 1 (MMP-1), MMP-9, monocyte chemoattractant protein 1 (MCP-1), MCP-3, granulocyte colony-stimulating factor (G-CSF), macrophage CSF (M-CSF), granulocyte-macrophage CSF (GM-CSF), tumor necrosis factor-α (TNF-α), interferon-γ (IFN-γ), galectin-1, platelet-derived growth factor-AA (PDGF-AA), and VEGF-A.

### Outcome measures

The primary endpoint was whether the recurrence intervals of ME were prolonged by anti-VEGF injections combined with STTA reported as percent of GRs. The secondary endpoint was a direct comparison of inflammatory cytokine concentrations in GRs and NRs. Additionally, we investigated inflammatory cytokine factors associated with GRs.

### Statistical analysis

Categorical data were assessed using Chi-square tests and continuous variables were assessed using Student’s *t*-tests. Comparisons of inflammatory cytokine concentrations in GRs and NRs were analyzed using non-paired *t*-tests. Inflammatory cytokines related to GRs were explored by logistic regression analysis after stepwise variable selection. Statistical analysis was performed using JMP Pro software ver. 15.0.0 (SAS Institute, Cary, NC). *P* values less than 0.05 indicated statistical significance.

## Results

### Patient characteristics

Forty-two eyes of 42 patients (24 men, 18 women) were investigated in this study (Table 1). Their mean age (± standard deviation) was 72 (± 9) years. BRVO was present in 17 eyes and CRVO was present in 25 eyes. Mean baseline best-corrected visual acuity (BCVA) (logMAR) was 0.33 (± 0.35) and mean baseline central subfield thickness (CST) was 445 (± 167) µm. Mean time from initial treatment to combination therapy was 1069 (± 631) days. The anti-VEGF drugs administered before combination therapy were aflibercept, ranibizumab, and bevacizumab in 23 eyes, 11 eyes, and 8 eyes, respectively. The number of anti-VEGF injections administered before combination therapy was 14 (± 10).

**Table 1:** Patient characteristics

Characteristic	*N* = 42
Age, years	72 (9)
Sex	
Male	24
Female	18
Type of RVO, number of eyes	
CRVO	25
BRVO	17
logMAR visual acuity before combination therapy	0.33 (0.35)
CST before combination therapy, µm	445 (167)
Time from initial treatment to combination therapy, days	1069 (631)
Type of anti-VEGF drug before combination therapy, number of eyes	
Aflibercept	23
Ranibizumab	11
Bevacizumab	8
Number of anti-VEGF injections administered before combination therapy	14 (10)

Values are shown as mean (standard deviation) or number

BRVO, branch retinal vein occlusion; CRVO, central retinal vein occlusion; CST, central subfield thickness

### Effectiveness of combination therapy

Twenty-six eyes (62%) showed GRs. Two of the eyes had no recurrence after combination administration. The average duration of the GR extension was 9.7 weeks. The clinical findings of the GR and NR groups are shown in Table 2. There were no significant differences in the clinical findings between the groups. Specifically, there were no significant differences in RVO type, logMAR visual acuity before combination therapy, CST before combination therapy, CST at recurrence in either group, type of anti-VEGF drug before combination therapy, number of anti-VEGF injections administered before combination therapy, history of photocoagulation, and mean time to recurrence before combination therapy.

**Table 2:** Clinical findings of GRs and NRs

Characteristic	GR group	NR group	*P* value
Eyes, number (%)	26 (62)	16 (38)	
Age, years	73 (9)	70 (9)	0.16*
Sex			0.93**
Male	15	9	
Female	11	7	
Type of RVO, number of eyes			0.76**
CRVO	15	10	
BRVO	11	6	
logMar visual acuity before combination therapy	0.30 (0.31)	0.36 (0.42)	0.60*
CST before combination therapy, µm	411 (159)	474 (198)	0.37*
CST at recurrence, µm	286 (163)	463 (208)	0.13*
Time from initial treatment to combination therapy, days	1188 (702)	874 (449)	0.11*
Type of anti-VEGF drug before combination therapy, number of eyes			0.21**
Aflibercept	12	11	
Ranibizumab	7	4	
Bevacizumab	7	1	
Number of anti-VEGF injections administered before combination therapy	16 (12)	12 (7)	0.62*
History of photocoagulation, number of eyes (%)	14 (54)	8 (50)	0.81**
Mean time to recurrence before combination therapy, weeks	11.9 (5.2)	11.4 (3.6)	0.73*
Extended interval (*n* = 24***), weeks	9.7 (6.7)		

Values are shown as mean (standard deviation) or number

*Paired t-test. **Chi-square test. ***2 eyes had no recurrence after combination therapy

BRVO, branch retinal vein occlusion; CRVO, central retinal vein occlusion; GR, good responders; NR, non-responders; RVO, retinal vein occlusion

### Inflammatory cytokines

**Fig. 1:**
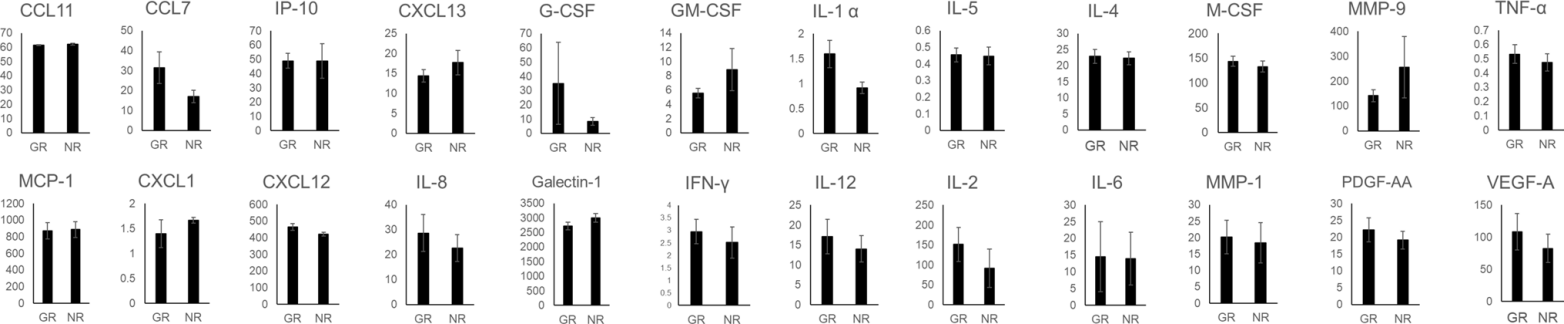
Inflammatory Cytokine Levels Inflammatory cytokines were not significantly different between the GR and NR groups. However, nominal logistic analysis showed that higher IL-1α (*p* = 0.016) and lower IL-5 (0.015), IL-6 (0.022), and galectin-1 (0.015) were significantly associated with prolonged recurrence intervals of ME

The concentrations of each inflammatory cytokine in the GRs and NRs are shown in Fig. 1. There were no significant differences in inflammatory cytokine levels between the groups. VEGF-A and IL-8 levels were higher in GRs than in NRs, but the difference was not significant; specifically, there were no significant differences in MCP-1, IL-6, and IP-10 levels. However, nominal logistic analysis showed that higher IL-1α (*P* = 0.016) and lower IL-5 (*P* = 0.015), IL-6 (*P* = 0.022), and galectin-1 (*P* = 0.015) were significantly associated with the extension of the time from injection to recurrence of ME.

Additional validation was performed in the aflibercept group. In GRs, aflibercept was observed in 12 eyes. The mean number of anti-VEGF injections administered before combination therapy was 21 (± 14), and the mean time to recurrence before combination therapy was 11 (± 4) weeks. In NRs, aflibercept was observed in 11 eyes. The mean number of anti-VEGF injections administered before combination therapy was 11 (± 5), and the mean time to recurrence before combination therapy was 12 (± 3) weeks. In GRs, there was no significant difference in galectin-1 concentrations between the aflibercept group and the other anti-VEGF drug group before steroid induction (2773 pg/ml vs. 2672 pg/ml, *P* = 0.63, non-paired *t*-tests).

### Safety

Complications included advanced cataracts in 4 eyes, for which cataract surgery was performed. No elevation of intraocular pressure was observed.

## Discussion

The results of this study revealed that combination anti-VEGF and STTA therapy was effective in 62% of eyes and the interval time to recurrence was extended by about 10 weeks. To our knowledge, there are no previous reports on the efficacy of combined anti-VEGF and STTA therapy for chronic RVO. As shown in Table 2, the mean time to recurrence before combination therapy was 11.9 weeks in GRs. Because the combination therapy could be extended for 9.7 weeks, we were able to halve the number of injections required. Even more surprisingly, 2 eyes had no recurrence after combination therapy at all. One patient had BRVO and had received 9 anti-VEGF drug injections as sole treatment with about 2 years having passed from the first treatment to the start of combination therapy. The other patient had CRVO and had received 6 anti-VEGF drug injections as sole treatment but had experienced repeated relapses within the 6 weeks prior to starting combination therapy. These results indicate that it may be important to suppress both VEGF and inflammation for chronic RVO.

A previous study reported that combination therapy had no effect on ME due to acute RVO in which symptom were present for < 4 months before the initial examination [19]. We speculate that VEGF may be one of the key factors underlying inflammation in the acute phase, and previous reports suggest that a vicious cycle of inflammation may underlie chronic RVO [3, 4]. We hypothesize that, as the disease becomes chronic, the role played by inflammation gradually increases. Therefore, we consider the timing of concomitant corticosteroid therapy initiation to be important. The clinical findings in the present study, including time from initial treatment to initiation of combination therapy, did not differ significantly between GRs and NRs, making it impossible to determine which patients would benefit from the combination therapy. In addition, the appropriate dosage timing was not clear because of differences in the timing of initiating combination therapy. Accordingly, we need to identify clinical findings that can indicate combination therapy as well as determine the appropriate timing for initiation of combination therapy.

The concentrations of inflammatory cytokines were not significantly different between the GR and NR groups. However, nominal logistic analyses revealed that higher IL-1α and lower IL-5, IL-6, and galectin-1 were significantly associated with the extension of the time from injection to recurrence intervals of ME.

IL-1α is a pro-inflammatory, pleiotropic cytokine involved in inflammation and immunity, and it is produced as a central driver of immune responses to tissue damage [20]. IL-1α has been shown in vitro and in vivo to directly increase the permeability of endothelial cells, and IL-1 has been shown in experimental models to be involved in the blood–retinal barrier breakdown seen in inflammatory eye diseases [21]. The mechanism of IL-1α in chronic RVO is unknown, but it may be strongly involved.

IL-5 is known for its role in eosinophil-associated diseases. IL-5 is the key cytokine involved in the regulation of blood and tissue eosinophils. In the human lung, IL-5 has been suggested to play an important role as a key regulator of angiogenic processes through signal transducer and activator of transcription 5 signaling [22]. However, there are no reports on the function of IL5 in retinal diseases, and thus further studies are needed to interpret the present results.

Another cytokine that can increase the permeability of endothelial cells is IL-6. It does this by inducing gap junctions to form between adjacent cells via actin filament rearrangement [23]. Previous studies reported elevated IL-6 and VEGF together in the vitreous and aqueous fluid of eyes with BRVO [24, 25]. Other studies have determined that elevated IL-6 is associated with the degree of ME [26–28]. In the present study, lower IL-6 was significantly associated with the extension of the time from injection to recurrence intervals of ME. Corticosteroids are effective for ME suppression. Although the pre-dose CST did not differ between the GR and NR groups and there was no significant difference in CST at relapse, it was smaller in GRs. The role of IL-6 in chronic RVO needs further study.

As for galectin-1, the present study showed significantly lower galectin-1 in RVO. In contrast, a previous study found elevated galectin-1 in diabetic retinopathy but not in RVO, suggesting that inflammation triggered by advanced glycation end products is the inducer of galectin-1 specifically in diabetic retinopathy [29]. Galectin-1 is known to interact with the N-glycans of VEGFR2 to increase VEGFR2 phosphorylation and activate its downstream signal transduction in endothelial cells in order to promote hypoxia-induced angiogenesis [30]. However, the relationship between galectin-1 and chronic RVO is currently unknown and requires further exploration.

This study analyzed chronic RVO patients who had received anti-VEGF therapy for more than 1 year and found that higher IL-1α and lower IL-5, IL-6, and galectin-1 were significantly associated with combined treatment effectiveness. These cytokines were measured immediately before starting the combined therapy. It is possible that cytokines other than IL-1α were suppressed by previous anti-VEGF therapy, and only IL-1α—which is deeply related to inflammation—showed high levels. There is scant research on inflammatory cytokines in chronic RVO, and thus further research is needed to elucidate the relationship between these inflammatory cytokines and combined treatment effectiveness.

### Limitations

This work has some limitations. First, it was a retrospective study. Second, the anti-VEGF drugs and the combination therapy timing were not standardized. Third, the number of cases is relatively small. In this study, discrepancies were observed between the results of individual factor analyses and those of the multivariable logistic regression analysis. These differences likely stem from the inherent nature of logistic regression, which accounts for the influence of multiple factors simultaneously, potentially revealing associations that may not be apparent in univariate analyses. In contrast, factors that appeared significant in the univariate analysis may lose significance when adjusted for other variables in the multivariable model. It is important to note that although logistic regression provides a more robust estimation of relationships by isolating the effects of individual factors, the lack of significance in univariate analysis suggests that such factors may not exert a strong or consistent influence. These considerations highlight the complexity of interpreting statistical associations and underscore the need for cautious interpretation of results.

Therefore, to confirm its utility, the effectiveness of the combination therapy for chronic RVO should be compared with anti-VEGF therapy alone in a prospective study with a larger number of cases.

## Conclusion

The combination of anti-VEGF drugs with STTA was effective for chronic RVO in 62% of patients. Moreover, treatment effectiveness was associated with a higher concentration of IL-1α and lower concentrations of IL-5, IL-6, and galectin-1. Inflammation may be strongly involved in chronic RVO, in addition to VEGF, and thus combination therapy with anti-VEGF drugs and corticosteroids is suggested to be a potential treatment.
